# Early Spondyloarthritis Clinic: Organizational Improvements in the Patient Journey

**DOI:** 10.3389/fmed.2022.833139

**Published:** 2022-05-24

**Authors:** Salvatore D'Angelo, Antonella Afeltra, Fabiola Atzeni, Elena Baldissera, Maurizio Caminiti, Francesco Ciccia, Maria Antonietta D'Agostino, Lorenzo Dagna, Gian Luca Erre, Franco Franceschini, Enrico Fusaro, Roberto Giacomelli, Elisa Gremese, Giuliana Guggino, Claudia Lomater, Ennio Lubrano, Angela Anna Padula, Giuseppa Pagano Mariano, Romualdo Russo, Piercarlo Sarzi Puttini, Raffaele Scarpa, Carlo Selmi, Enrico Tirri, Stefano Ferri, Florenzo Iannone

**Affiliations:** ^1^Dipartimento Regionale di Reumatologia, Azienda Ospedaliera Regionale (A.O.R.) San Carlo, Potenza, Italy; ^2^Unità Operativa Complessa (U.O.C.) Immunoreumatologia, Campus Bio-Medico, Rome, Italy; ^3^U.O.C. Reumatologia, AOU G. Martino, Messina, Italy; ^4^Unità Operativa (U.O.) Immunologia, Reumatologia, Allergologia e Malattie Rare, Istituto di Ricovero e Cura a Carattere Scientifico (I.R.C.C.S.) Ospedale S. Raffaele, Milan, Italy; ^5^U.O. Reumatologia, Grande Ospedale Metropolitano Bianchi-Melacrino-Morelli, Reggio Calabria, Italy; ^6^U.O.C. Reumatologia, Azienda Ospedaliera Universitaria (A.O.U.) Vanvitelli, Naples, Italy; ^7^U.O.C. Reumatologia, Policlinico Universitario A. Gemelli-I.R.C.C.S., Università Cattolica del Sacro Cuore, Rome, Italy; ^8^Struttura Complessa (S.C.) Reumatologia, A.O.U. Sassari, Sassari, Italy; ^9^U.O.C. Reumatologia e Immunologia Clinica, Azienda Socio Sanitaria Territoriale (A.S.S.T.) Spedali Civili, Brescia, Italy; ^10^S.C. Reumatologia, A.O.U. Città della Salute e della Scienza, Turin, Italy; ^11^U.O.C. Reumatologia, A.O.U. P. Giaccone, Palermo, Italy; ^12^Struttura Semplice Dipartimentale (S.S.D.) Reumatologia, Azienda Ospedaliera (A.O.) Ordine Mauriziano, Turin, Italy; ^13^Unità Operativa Semplice Dipartimentale (U.O.S.D.) Reumatologia, Presidio Ospedaliero (P.O.) Cardarelli, Campobasso, Italy; ^14^U.O.S. Reumatologia, Azienda Ospedaliera di Rilievo Nazionale (A.O.R.N.) Cardarelli, Naples, Italy; ^15^U.O. Reumatologia, Azienda Socio Sanitaria Territoriale Fatebenefratelli (A.S.S.T. F.B.F.) Sacco, Milan, Italy; ^16^U.O. Reumatologia e Riabilitazione Reumatologica, A.O.U. Federico II, Naples, Italy; ^17^U.O. Reumatologia e Immunologia Clinica, Istituto Clinico Humanitas – I.R.C.C.S., Rozzano, Italy; ^18^Dipartimento di Scienze Biomediche, Humanitas University, Milan, Italy; ^19^U.O.S.D. Reumatologia, Presidio Ospedaliero San Giovanni Bosco (P.O. S.G.), Naples, Italy; ^20^EY Advisory Italy, Milan, Italy; ^21^Dipartimento di Emergenza e Trapianto d'Organi, U.O.C. Reumatologia Universitaria, Clinica Reumatologica, Scuola di Specializzazione in Reumatologia, Bari, Italy

**Keywords:** spondyloarthritis, Early SpA Clinic, rheumatology, patient journey, early diagnosis, hospital management, hospital organization

## Abstract

Spondyloarthritis are chronic inflammatory diseases affecting spine, peripheral joints and enthesis, as well as extra-articular sites (bowel, eyes, skin). Diagnosis of spondyloarthritis often is slow and requires a multidisciplinary approach. The “Early SpA Clinic” project aimed at improving the patient care and journeys, by solving some organizational issues existing in Rheumatology Clinics. The “Early SpA Clinic” involved 19 Italian Rheumatology Centers using in-depth organizational analyses to identify areas for improvement. From the results of the analyses, some organizational solutions were suggested, and their impact measured at the end of the project through specific KPI. With the implementation of the suggested organizational solutions, Centers achieved relevant results, positively impacting on all the phases of the patient journey: decrease in waiting lists (−23%) and in the time length to transit the Center (−22%), increase in the percentage of new diagnoses (+20%), in the saturation of outpatient clinic capacity (+16%), and in the patient satisfaction (+4%). Centers involved in the “Early SpA Clinic” implemented several organizational actions based on an overall assessment of their activities and on solutions that required no additional resources. Overall, the Centers achieved the “Early SpA Clinic” objectives in terms of better management of resources, personnel, spaces, equipment, in relation to the volumes of patients.

## Introduction

### Rationale for the “Early SpA Clinic”

The family of seronegative spondyloarthritis (SpA) includes a heterogeneous group of diseases linked by typical articular and extra-articular manifestations, characteristic signs to instrumental investigations and lack of autoantibodies. According to the Italian Society of Rheumatology, diseases belonging to the SpA group can be classified in forms with prevalent axial involvement (Radiographic Axial SpA and Non-Radiographic Axial SpA), predominant peripheral involvement (Psoriatic Arthritis, Entero-pathic SpA, Reactive SpA) and other undifferentiated forms (1).

SpA should be considered as a systemic disease, with extra-articular involvement seen in more than half of the patients. The involvement of the gut (e.g., Inflammatory Bowel Disease), eye (e.g., anterior uveitis) and skin (e.g., psoriasis, pyoderma gangrenous, erythema nodosum) often precedes the onset of joint manifestations (2, 3).

Because of its multi-faceted appearance, SpA are rarely diagnosed promptly. Consequently, there may be a delay in SpA diagnosis and in starting a pharmacological treatment (3, 4). Diagnostic delay in SpA identification represents the main concern related with these diseases, as it may raise direct and indirect costs, it impacts the prognosis and undermines the therapeutic potential (4–6). In SpA as well as in all rheumatic diseases there is a “window of therapeutic opportunity,” namely a time period that runs from the onset of the first symptoms to the moment where the structural, irreversible damage begins (4, 5, 7). During this timeframe, any drug has a chance of success greater than if it is administered later. The greatest diagnostic delay is observed in the Axial SpA, where patients wait even 10 years on average from the onset of the disease to diagnosis and therapy (4, 6, 7).

A factor contributing to late identification of these diseases is the lack of specific biomarkers, which could help in early diagnosis. In addition, referral is more complex in SpA than in other rheumatic diseases such as Rheumatoid Arthritis as it requires a multichannel approach (i.e., the involvement of several specialists) (7–9). The above issues reflect some of the main unmet needs of SpA diseases, which the Early SpA Clinic, a project providing an organizational model, useful for improving the performance of the healthcare facility tried to face and solve.

### Objectives

The “Early SpA Clinic” aimed to improve the patient care and journeys in Rheumatology, and specifically in SpA, inspired by the principle of patient centrality and care optimization, through an early care and efficient use of organizational resources. The definition of lean pathways to minimize waiting lists and improve patients' experience within the Center were additional objectives pursued.

## Materials and Methods

### Involved Centers and Phases

**Figure 1:**
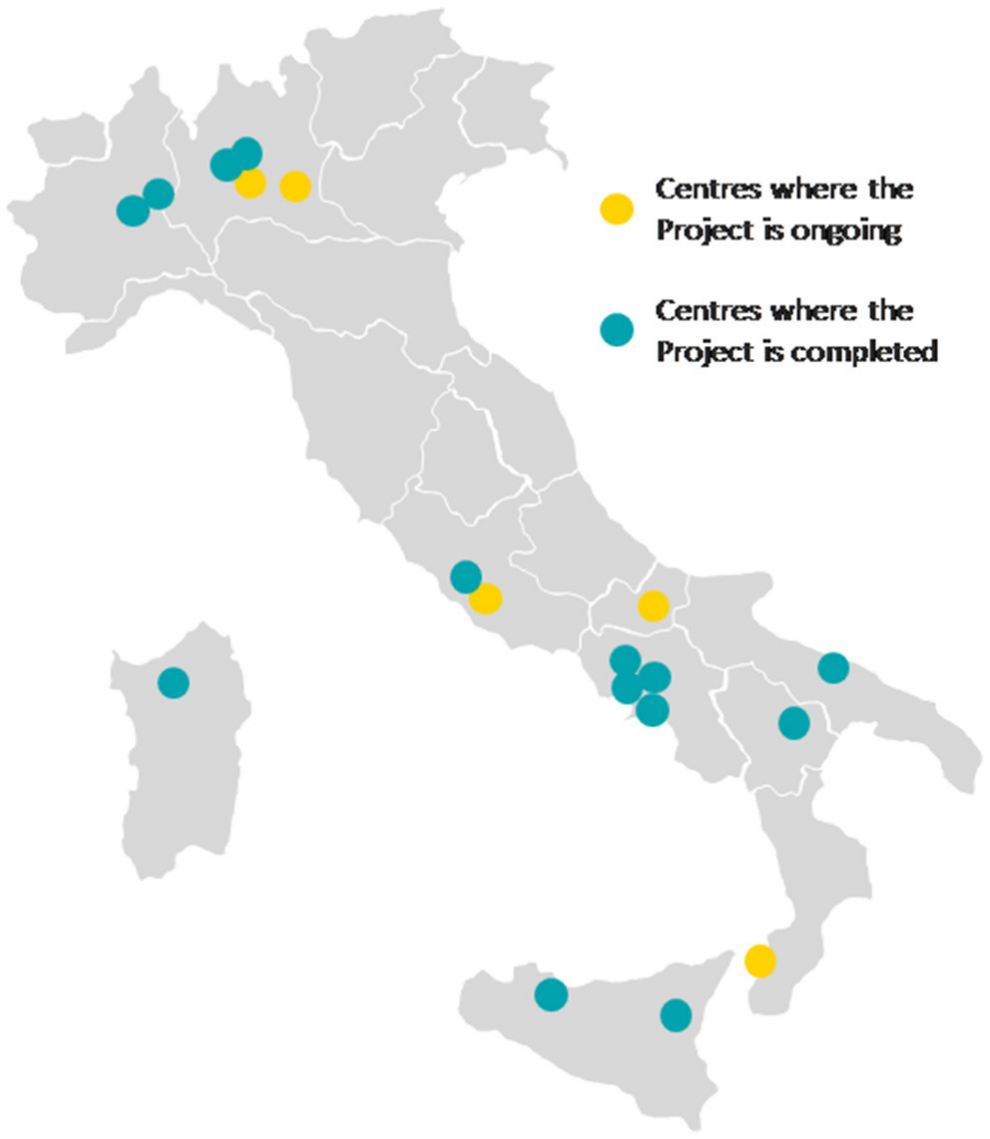
Map of the centers involved in the “Early SpA Clinic” project.

The “Early SpA Clinic” project involved 19 Rheumatology Clinics, the majority being public Hospitals in the South of Italy, and it was completed in 14 Centers at the time of writing (Figure 1). Based on the project progress, some data are available for 17 Centers.

**Figure 2:**
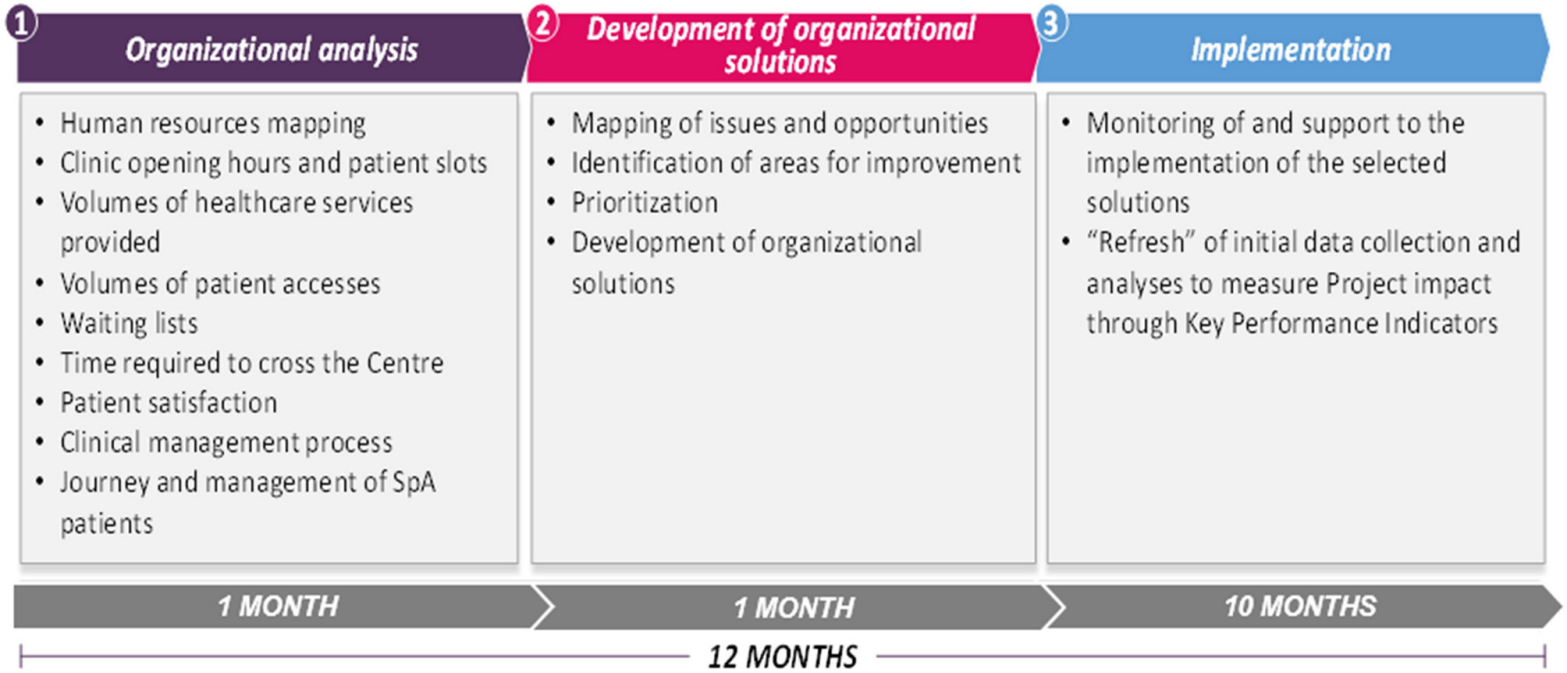
Overview of the “Early SpA Clinic” phases and activities.

The “Early SpA Clinic” project had a total duration of 12 months, and it is divided into three main phases with different objectives and planned activities, as detailed in Figure 2.

The initial phase consisted of collection and analysis of organizational data relating to the capacity of each Rheumatology Clinic, the volumes of services provided and of attending patients, and the activities offered.

The second phase aimed to map issues and opportunities for the Center based on the results of the analyses performed. Issues and opportunities were ranked according to their estimated impact, probability of occurrence and measurability.

The various issues and opportunities were then prioritized and connected to specific organizational solutions and proposed to the Center to address the identified areas for improvement. The solutions were clustered by type: a) same-resource solutions, when they concerned re-organizations and improvements with available resources, involving activities that could be implemented directly by the Rheumatology Center; b) additional resource solutions, if they required the Management's approval or additional resources to be implemented.

The final phase lasted from 9 to 10 months, and it aimed to support the implementation of the organizational solutions proposed. In the last month of the “Early SpA Clinic,” the analyses carried out in the first month were updated, allowing to measure the project impact on specific Key Performance Indicators (KPIs) identified for each phase of the patient journey. Regarding the access phase, the identified KPIs were the number of days of waiting lists, particularly for a first visit, and the time required by a patient to transit the Center (i.e., from the moment the patient enters the hospital, registers, receives the visit and exits from the hospital). Regarding the diagnosis phase, the “Early SpA Clinic” measured changes in the percentage of new diagnoses and in the saturation of outpatient clinic capacity. In addition, it verified the existence of collaboration agreements with other hospital services, such as Radiology. Finally, the “Early SpA Clinic” measured the patient satisfaction as a KPI for the management and follow-up phase.

### Organizational Analyses

The first type of analysis assessed the Rheumatology Clinic capacity in terms of human resources (e.g., clinicians, other medical, nursing and ad-ministrative staff), to understand their allocation, also considering their contract, role and shifts. In addition, the analysis covered the clinic opening hours and patient slots based on the agendas, with a possible distinction between the Hospital booking agenda (usually dedicated to first visits) and the clinicians' agendas (usually dedicated to follow-ups). The number of no-show patients was also recorded for each clinic. The combination of this data allowed the evaluation of, on the one hand, the clinic potential resource capacity, and on the other hand, the saturation of visit slots, comparing the weekly slots planned with the actual number of patients examined.

A second type of analysis assessed the volumes of outpatient services provided in previous years.

Through direct interviews with the Hospital booking center representatives, as well as through the analysis of the internal planning agendas, the waiting lists for different types of visits, for each outpatient clinic, were also traced.

The analysis of the time required by a patient to transit the Center (i.e., from the moment she enters the Hospital, registers, receives the visit and exits from the Hospital) tracked the patient's average time spent at the registration desk, in the clinic waiting room and in the clinician's office, thus highlighting the total time spent within the Center, comparing it to the actual time needed to receive the healthcare service.

The clinical management process was analyzed in terms of actors involved and related activities performed in the various phases of the patient journey, highlighting possible issues (e.g., lack of digital supports such as electronic medical records) or areas for improvements.

The patient satisfaction analysis consisted of a questionnaire directly filled-in by the patients based on a Likert scale (10). Each question required an evaluation of the degree of satisfaction from 1 (very dissatisfied) to 7 (very satisfied) of the following dimensions: waiting times, information received before and after the visit, quality of the Center structure, quality of the received services, quality of the Rheumatology Clinic care management (e.g., perceived adequacy of the visit duration).

Additional analyses assessed the clinical journey and management of Early SpA patients, as a focus of the project. Specifically, an analysis of the patient management model was performed, designing the patient journey and assessing the duration of each phase (e.g., from first visit to diagnosis), the different activities carried out by the Center staff, the spaces and tools involved, to highlight possible issues or areas for improvement.

Finally, for organizational purposes, the “Early SpA Clinic” collected data on patient accesses divided by disease (i.e., Non-Radiographic Axial SpA, Ankylosing Spondylitis, Psoriatic Arthritis, Rheumatoid Arthritis, Systemic Lupus Erythematosus, Scleroderma, Vasculitis, Other), by type of patients (patients in follow-up, patients without a diagnosis and patients with a diagnosis made in another Center) and by type of outpatient clinics.

## Results

Following the organizational analyses results, the “Early SpA Clinic” identified criticalities and specific areas for improvement in each phase of the patient journey (i.e., access, diagnosis, management and follow-up), in each Center and shared improvement proposals and related results. This section reports the suggested same-resource solutions only, and results achieved through these types of activities. The “Early SpA Clinic” enrolled 19 Centers overall: different set of data are available for 17 centers, among those, 14 had completed the project at the time of writing. In some cases, data collection was not concluded due to an early interruption of the project or to obstacles related to the Covid-19 pandemic.

**Figure 3:**
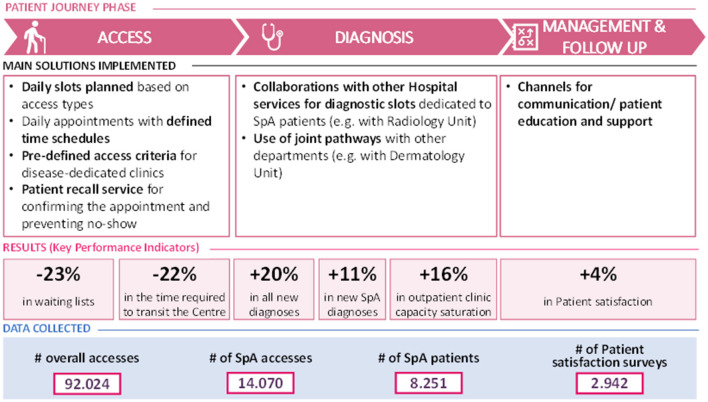
Synthetic overview of the “Early SpA Clinic” results.

A synthetic overview of the main organizational solutions implemented for each phase of the patient journey, with related “Early SpA Clinic” results and data collected is presented in Figure 3.

### Access–Criticalities at Baseline

Among the 19 centers selected for the project, as far as the access phase is concerned, 11 Centers did not have daily slots specifically dedicated to first visits, or if they did, they were often insufficient considering the number of patient accesses. A disease-dedicated outpatient clinic was not present in 7 centers, or if they did, there were no criteria established for a direct access thereto. Consequently, even if patients were already diagnosed in other Centers, they were referred to the “General Rheumatology” Outpatient Clinic when accessing the Center for the first time. In 6 Centers, a specific hourly schedule for daily appointments was not defined (i.e., the patients received a booking confirmation with a wide time slot). In 5 Centers, there were either no (or insufficient) daily slots dedicated to emergencies. Finally, 14 Centers did not have any tools for patient recall, so that in case of no-show patients it was impossible to reallocate vacant slots. These issues mostly impacted on the length of waiting lists for a first visit, equal to 6 months on average (with a minimum of 28 to a maximum of 455 days).

### Access–Improvement Proposals and Related Results

Considering the identified issues, some same-resource solutions have been suggested, consisting in a revision of the clinic daily slot planning, to explicitly distinguish between the slots dedicated to first visits from those dedicated to follow-ups, consistently with the types of registered accesses. The rationale behind this suggestion was based on the different duration of each type of visit, being a follow-up usually shorter than a first visit. This approach aimed to better allocate (and potentially increase) the number of patients examined per week.

In 5 Centers, the analyses led to suggest planning daily appointments based on defined timetables, or at least to identify a time range, and to communicate it to the patients. In 3 Centers, the “Early SpA Clinic” suggested to establish predefined criteria to access disease-dedicated outpatient clinics, so that already diagnosed patients could be sent immediately to the most relevant specialist (i.e., with appropriate competencies). Finally, the set-up of an operator service of patient recall was suggested in 9 Centers, so that no-show patients could be monitored in advance.

**Figure 4:**
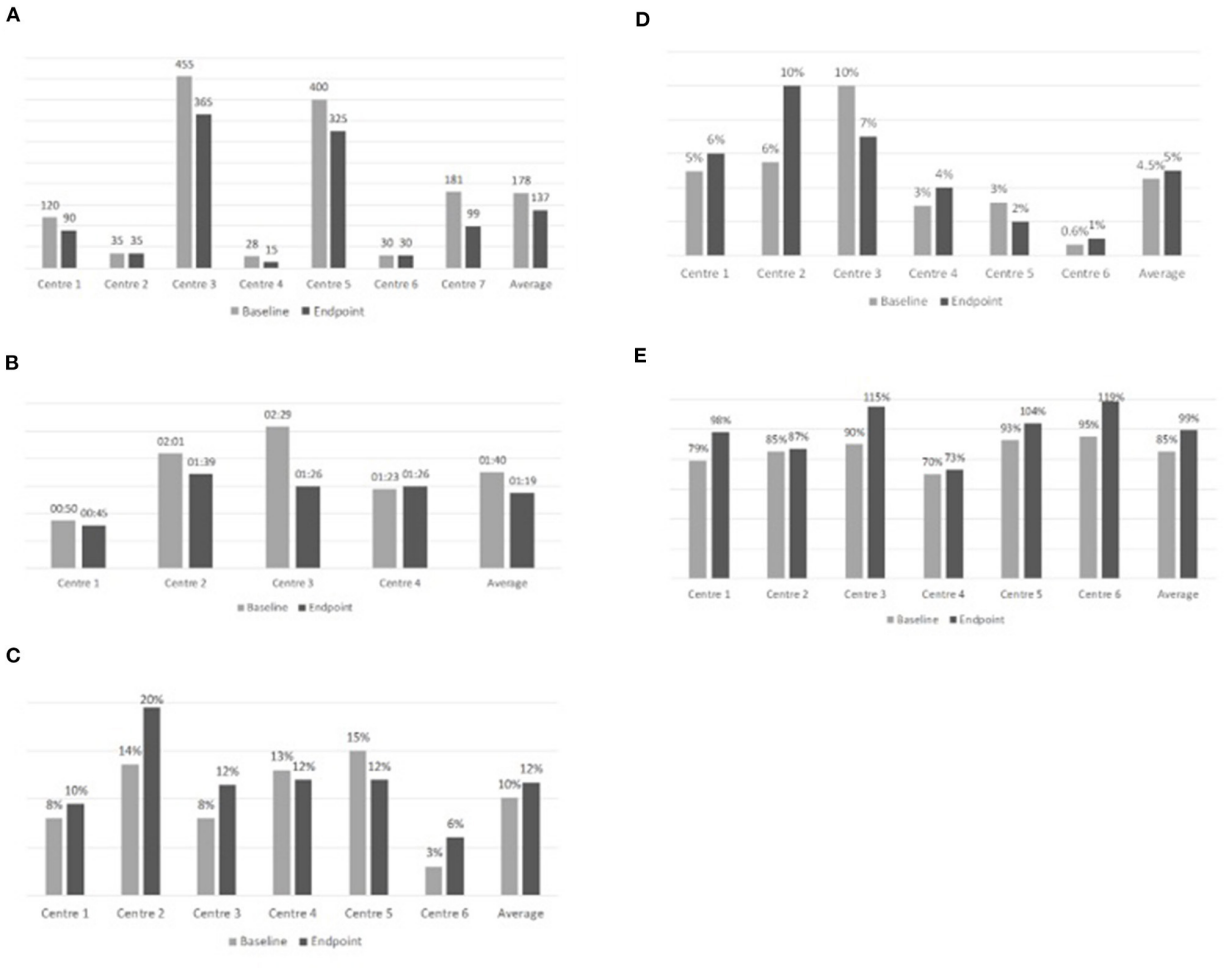
Overview of results achieved during the “Early SpA Clinic” project after implementation of organizational solutions. **(A)** Waiting lists (days). **(B)** Time required by a patient to transit the Center (hours). **(C)** Percentage of new diagnoses of Rheumatology patients (%). **(D)** Percentage of new SpA diagnoses (%). **(E)** Saturation of production capacity (%).

At the end of the project, waiting lists were reduced by 23%, from 178 to 137 days on average, in all Centers (N=7) that adopted these solutions and where it was possible to compare the KPI at the beginning with the KPI at the end of the “Early SpA Clinic” project (Figure 4).

Waiting lists were already short and did not register any improvement in Center 2 and 6, also due to a partial implementation of the suggested solutions.

The minimum waiting time diminished from 28 to 15 days, while the maximum waiting time diminished from 455 to 365 days (Box 1).

Box 1 **A practical case.**

**Criticalities at baseline**

The analyses highlighted the following issues related to the access phase in a Public Hospital:

- Long waiting lists (equal to 120 days) to access the General Rheumatology Clinic due to limited available slots for first visits, emergencies, and first visits of patients already diagnosed in another Center
- Impossibility to predict daily patient flows as appointments are not scheduled within a precise timeframe

**Improvement proposals and related results**

The solutions suggested and implemented throughout the project related to:

- The set-up of slots dedicated to patients with urgent prescriptions at the end of the day
- The scheduling of appointments within defined timeframes, equally dividing the number of patients among clinicians

At the end of the project, the General Rheumatology Clinic recorded a significant reduction (−25%) in the waiting lists, that reduced from 120 to 90 days.

**Figure 5:**
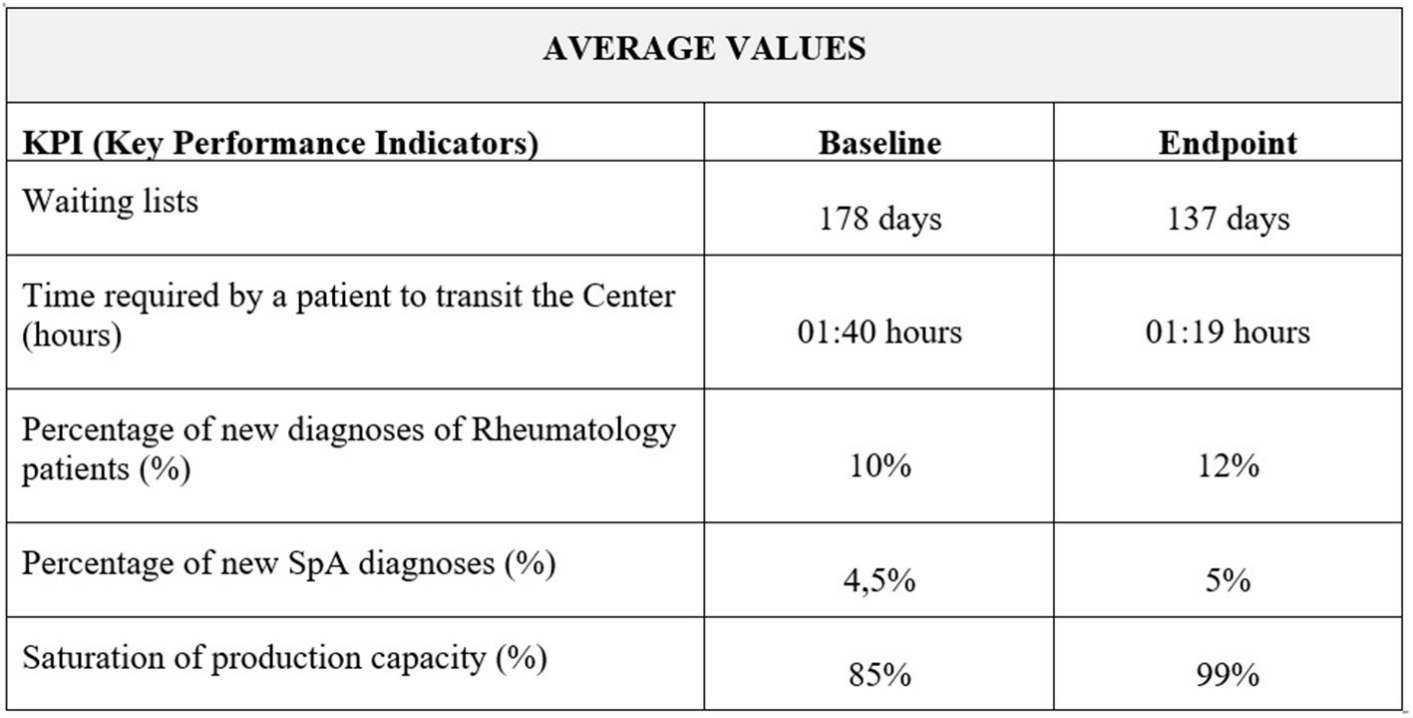
Overview of results (total average values).

In addition, the “Early SpA Clinic” led to a reduction in the time required by a patient to transit the Center, in all the Centers (N=4) that implemented these solutions and where it was possible to compare the KPI at the beginning with the KPI at the end of the “Early SpA Clinic.” In these Centers, the time required by a patient to transit the Center diminished by 22%, passing on average from 1h40 to 1h19. The minimum time required by a patient to transit the Center has diminished from 0h50 to 0h45, while the maximum passed from 2h29 to 1h39 (Figure 5).

The time required by a patient to transit the Center was already low in Center 4, where it registered a small increase at the end of the “Early SpA Clinic.” However, given that endpoint data were collected during the Covid-19 pandemic, this result could be deemed as satisfactory, considering the need for complying with safety measures (e.g., sanitation of the clinics) to contain the spread of the pandemic (Box 2).

Box 2 **A practical case.**

**Criticalities at baseline**

The analyses conducted in a Public Hospital highlighted the impossibility to predict daily patient flows, as appointments are not scheduled within a precise timeframe.

**Improvement proposals and related results**

The solutions suggested and implemented throughout the study related to the scheduling of appointments within defined timeframes and communicated in advance to the patient. At the end of the study, the time required by a patient to transit the Center reduced by 42% when compared to data collected at the beginning of the study, going from 2h29 to 1h26.

### Diagnosis–Criticalities at Baseline

As far as the diagnosis phase is concerned, out of 19 Centers, in 14 Centers there were no slots for diagnostic investigations dedicated to rheumatology patients. In 10 Centers, the Radiology and Laboratory services were reserved to hospitalized patients, while in 5 Centers it was impossible to perform the entire di-agnostic process within the Center due to a lack of specific diagnostic instruments.

The most evident impact of these issues resulted in the difficulty of completing the diagnostic process in a short timeframe. Another effect was identified in the lack of a SpA diagnostic path integrated with other Departments for in-depth investigations on comorbidities.

### Diagnosis–Improvement Proposals and Related Results

Considering these issues, the proposed same-resource solutions suggested the definition of collaboration agreements with other Hospital services, such as Radiology, aimed at setting up specialist diagnostic tests (in 9 Centers) and visit slots dedicated to multidisciplinary specialist advice (in 8 Centers).

At the end of the “Early SpA Clinic,” the percentage of new diagnoses of Rheumatology patients registered an average increase equal to 20%, passing from 10 to 12%, in all Centers (*N* = 6) that implemented these solutions and where it was possible to compare the KPI at the beginning with the KPI at the end of the “Early SpA Clinic” (Figure 4).

The percentage of new diagnoses of Rheumatology patients did not register any improvement in Center 4 and 5 since the implementation of the suggested solutions was not completed due to the outbreak of the Covid-19 pandemic.

With specific reference to SpA patients, the percentage of new diagnoses increased on average by 11%, passing from 4.5 to 5% (Figure 4).

The percentage of new SpA diagnoses did not register any improvement in Center 3 and 5 since the implementation of the suggested solutions was not completed.

Following the new diagnoses, at the end of the “Early SpA Clinic” the same 6 Centers also registered an increase in the saturation of production capacity (i.e., in the number of slots planned and then actually used each week, thus indicating a potential increase in the number of patients per week). On average, the percentage of production capacity increased by 16% (from 85 to 99%) (Figure 5). Refer to Box 3 for the practical case.

Box 3 **A practical case.**

**Criticalities at baseline**

The organizational analyses highlighted the lack of slots for multidisciplinary medical examinations, as well as the lack of an officially recognized clinical journey in a Center.

**Improvement proposals and related results**

The solutions suggested and implemented throughout the project related to the set-up of an outpatient clinic shared with the Dermatology Department, where multidisciplinary collab-orations were activated on a monthly basis for specific diagnosis. At the end of the study, the percentage of SpA new diagnoses increased by 37%, while the saturation of production capacity increased by 25%.

### Management and Follow-Up–Criticalities at Baseline

Finally, as far as the management and follow-up phase is concerned, among the 19 Centers involved, in 15 Centers a drop-out monitoring system was lacking. In 3 Centers there were no communication channels for contacting and educating patients. The most evident impact was reflected in the failure to monitor the drop out (and consequently the patient's compliance with disease-related follow-up schedules), as well as in a poor communication and support toward patients.

### Management and Follow-Up–Improvement Proposals and Related Results

Considering the identified issues, the same-resource solutions suggested related to enhanced communication channels and support for patient education.

At the end of the “Early SpA Clinic,” only one Center was able to implement a solution related to the management and follow-up phase, by setting up communication channels dedicated to patients (or to their healthcare practitioner) to request support in the therapy or disease management. This solution granted a moderate increase (+3.6%) in the patient satisfaction registered by the Center, concerning specifically to the area of “information provided by the Center.” The increase of the patient satisfaction is calculated as an incremental change in the overall patient satisfaction level, registered at the beginning of the project (baseline) and later after the solutions proposed were implemented (endpoint). The moderate result could be due to an already high initial patient satisfaction score (5.6/7).

## Discussion

By means of the implementation of some of the suggested organizational solutions, the involved Centers achieved relevant results, positively impacting on all the phases of the patient journey. Overall, the “Early SpA Clinic” led to a decrease in waiting lists by 23% and in the time to transit the Center by 22%, an increase in the percentage of new diagnoses of rheumatology patients (+20%), in the saturation of outpatient clinic capacity (+16%), and in the patient satisfaction (+4%).

From a literature review, best practices and strategies carried out in other Countries regarding the management of patients with Spa have been analyzed.

The analysis revealed that in other countries there is the need for integrating rheumatology nurses into the patient care pathway. In Spain, for instance, a study was conducted to propose the integration of rheumatology nurses in the patient pathway of Axial SpA patients (e.g., e-consultations led by rheumatology nurse for monitoring stable patients) (11). In France, the helpfulness of the role of rheumatology nurses has been confirmed by a research study that pointed out benefits of a nurse led program about the self-management of the diseases in young people with Axial SpA (12).

Moreover, Santos-Moreno (13) performed a study to standardize the care of patients with SpA in Latin America. Based on the experience of three Centers of excellence for SpA, the study proposed a patient-centric and multidisciplinary care model that measures the quality of care through some indicators, such as access to diagnostic support and multidisciplinary team involvement, access to treatment and quick responses to the needs of the patient, patient education (13).

The “Early SpA Clinic” implemented in Italian Rheumatology Centers represents a tailored and structured organizational strategy compared to other adopted care models.

The “Early SpA Clinic” tried to help Rheumatology Centers in setting up specific organizational solutions to target the main issues in diagnosis and follow-up of SpA patients. The results achieved are promising, especially in terms of shorter waiting lists, shorter times required to transit the Center, higher number of slots dedicated to diagnosis, and saturation of outpatient clinic capacity.

However, the study has some limitations, due to the small number of Centers for which it was possible to measure the organizational solution impact through data collection both at the beginning and at the end of the project. A major obstacle to data collection conclusion is related to the Covid-19 pandemic, especially in the first five months of 2020.

In addition, it is worth mentioning that the “Early SpA Clinic” also considered the Referral area as a first phase of the patient journey. However, the organizational solutions and the results achieved in this area are not described, as they are rarely of the same-resource type and most of activities require the involvement of third parties outside the Rheumatology Center. Referral issues and solutions within the Centers will be targeted in future research studies, whose objectives will be to cover the entire patient journey to further increase care standards, providing for the involvement of local/territorial healthcare providers, with a view of improving the appropriateness of referrals by General Practitioners or local specialists, setting up a referral triage and direct referral systems. The relevance of referral, possible instruments, and strategies on which future studies will be based are widely discussed in the literature (13–17).

Given SpA-related multi-organ involvement, complexity, and difficulties as well as delays in the diagnosis and treatment phase, there is a strong need for: (a) anticipating and focusing on diagnosis, possibly granted by a clear distinction between slots dedicated to first vs. follow-up visits; (b) a multidisciplinary approach, granted by outpatient clinics shared among different Departments (e.g., Rheumatology and Dermatology) or at least visit slots dedicated to multidisciplinary consultancies; (c) involving the patients, by establishing communication channels and possibly monitoring the drop-out to control patients' compliance with follow-up timings.

In addition to the results achieved, the implementation of the “Early SpA Clinic” generated additional benefits for Rheumatology Centers.

Firstly, gathered data and related analyses raised awareness on the volumes of outpatient activities performed by the Rheumatology Clinics, and on the volumes of patients treated, which was not always clear to each Center.

The “Early SpA Clinic” also brought benefits related to a better management of the trade-off between waiting lists and internal efficiency (available resources vs. volumes of activity), a more efficient synchronization of supply with demand, a better management of personnel, spaces and equipment, in relation to the volumes of patients. The achievement of organizational effectiveness has also made it possible to innovate the care models, to increase the attractiveness of each Center and to promote its sustainability over time.

Furthermore, through the organizational analyses carried out by a third and independent party, the “Early SpA Clinic” has allowed the Rheumatology Centers to highlight the activities performed, the results achieved, and to deal with the Management on possible needs in terms of resources (as evidenced by the study).

Finally, the “Early SpA Clinic” project and the results achieved made it possible to increase the visibility of the Rheumatology Departments within the Centers, and to initiate collaborations and synergies between existing out-patient services.

## Conclusions

By means of the “Early SpA Clinic,” the Rheumatology Centers involved implemented several improvements, based on an overall assessment of their activities and on solutions that required no additional resources.

The “Early SpA Clinic” highlighted there are some organizational solutions that could be set up by Rheumatology outpatient clinics with no or very limited effort, to ensure the SpA patient early care and a smooth and inclusive patient-centered journey. In addition, these organizational solutions can bring tangible and immediate improvements (i.e., within 12 months) to the Center performance.

From the analysis of organizational solutions for SpA patients implemented at an international level, it has been revealed that the organizational model adopted in the “Early SpA Clinic” project could represent an example of a standard organizational model. However, taking into account Health Systems' differences among Countries is fundamental. The “Early SpA Clinic” project has been implemented in Italian Rheumatology Centers considering peculiarities of the Italian National Health System. In order to be applied as an example for similar situations, an adaptation based on both context and Health Systems' features in other Countries is required.

## Data Availability Statement

The original contributions presented in the study are included in the article/supplementary material, further inquiries can be directed to the corresponding author.

## Author Contributions

SD'A and FI contributed to the writing, editing and overall revision of the paper, as well as in the identification of organizational solutions. AA, FA, EB, MC, FC, MD'A, LD, GLE, FF, EF, RG, EG, GG, CL, EL, AAP, RR, PSP, RS, CS, and ET contributed to the identification and validation of the organizational solutions. All authors read and approved the final manuscript.

## Funding

This study received funding from Novartis Farma SpA, who involved EY Advisory SpA for data collection, analyses, project management, and implementation. The funder was not involved in the study design, collection, analysis, interpretation of data, the writing of this article, or the decision to submit it for publication.

## Conflict of Interest

The authors declare that the research was conducted in the absence of any commercial or financial relationships that could be construed as a potential conflict of interest.

## Publisher's Note

All claims expressed in this article are solely those of the authors and do not necessarily represent those of their affiliated organizations, or those of the publisher, the editors and the reviewers. Any product that may be evaluated in this article, or claim that may be made by its manufacturer, is not guaranteed or endorsed by the publisher.

## References

[1] D'Angelo S Bajocchi G Cauli A Conti F Cutolo M Scarpa R. Malattie infiammatorie articolari e periarticolari. Reumatismo. (2019) 71:11–3. Available online at: https://www.reumatologia.it/obj/files/AttiCongressi/REUMA_SUPPL_2_2019_ita_LOWRES.pdf

[2] Churcher L Chan CH Badley EM. Chronic back problems and labor force participation in a national population survey: impact of comorbid arthritis. BMC Public Health. (2013) 13:326. 10.1186/1471-2458-13-326 23575216 PMC3626871

[3] van der Horst-Bruinsma IE Nurmohamed MT. Management and evaluation of extra-articular manifestations in spondylo-arthritis. Ther Adv Musculoskelet Dis. (2012) 4:413–22. 10.1177/1759720X12458372 23227118 PMC3512172

[4] Poddubnyy D Sieper J. Diagnostic delay in axial spondyloarthritis - a past or current problem? Curr Opin Rheumatol. (2021) 33:307–12. 10.1097/BOR.0000000000000802 33882509

[5] Mennini FS Viti R Marcellusi A Sciattella P Viapiana O Rossini M. Economic evaluation of spondyloar-thritis: economic impact of diagnostic delay in Italy. Clinicoecon Outcomes Res. (2018) 10:45–51. 10.2147/CEOR.S144209 29391817 PMC5768184

[6] D'Angelo S Malavolta N Scambi C Salvarani C Caso F Tirri E. Quality of life and therapeutic management of axial spondyloarthritis patients in Italy: a 12-month prospective observational study. Clin Exp Rheumatol. (2021) 39:961–9. 33427620 10.55563/clinexprheumatol/dz0xrd

[7] Zwolak R Suszek D Graca A Mazurek M Majdan M. Reasons for diagnostic delays of axial spondyloar-thritis. Wiad Lek. (2019) 72:1607–10. 10.36740/WLek201909102 31586971

[8] Rizzello F Olivieri I Armuzzi A Ayala F Bettoli V Bianchi L. Multidisciplinary Management of Spondyloarthritis-Related Immune-Mediated Inflammatory Disease. Adv Ther. (2018) 35:545–62. 10.1007/s12325-018-0672-6 29516409 PMC5910456

[9] Olivieri I Accorinti M Abicca I Bisceglia P Cimino L Latanza L. CORE Study Group. Standardization of red flags for referral to rheumatologists and ophthalmologists in patients with rheumatic diseases and ocular involvement: a consensus statement. Rheumatol Int. (2018) 38:1727–34. 10.1007/s00296-018-4094-1 29961101

[10] Joshi A Kale S Chandel S. Likert Scale: Explored and explained. Curr J Appl Sci Technol. (2015) 7:396–403. 10.9734/BJAST/2015/14975

[11] Carrillo I López-Pineda A García-Díaz S López A Muntalà LV Juanola X. Propuestas para la incorporación del rol de enfermería en la certificación de unidades de espondiloartritis axial. Revisión bibliográfica y consenso entre expertas. Reumatol Clín. (2021) 11:184–192. 10.1016/j.reuma.2021.09.005

[12] Molto A Gossec L Poiraudeau S Claudepierre P Soubrier M Fayet F. Evaluation of the impact of a nurse-led program of patient self-assessment and self-management in axial spondyloarthritis: results of a prospective, multicentre, randomized, controlled trial (COMEDSPA). Rheumatology. (2021) 60: 888–95. 10.1093/rheumatology/keaa480 33063096

[13] Santos-Moreno P Baraliakos X García-Salinas R. Engagement process for patients with spondyloarthritis: PANLAR early SpA clinics project — centers of excellence. Clin Rheumatol. (2021) 40:4759–66. 10.1007/s10067-021-05806-4 34273002

[14] Gudu T Jadon DR. Multidisciplinary working in the management of axial and peripheral spondyloarthritis. Ther Adv Musculoskelet Dis. (2020) 12:1–14. 10.1177/1759720X20975888 33354231 PMC7734487

[15] Felice C Leccese P Scudeller L Lubrano E Cantini F Castiglione F. Italian SpA-IBD Expert Panel Group. Red flags for appropriate referral to the gastroenterologist and the rheumatologist of patients with inflammatory bowel disease and spondyloarthritis. Clin Exp Immunol. (2019) 196:123–38. 10.1111/cei.13246 30554407 PMC6422654

[16] Poddubnyy D van Tubergen A Landewé R on behalf of the Assessment of SpondyloArthritis international Society (ASAS). Development of an ASAS-endorsed recommendation for the early referral of patients with a suspicion of axial spondyloarthritis. Ann Rheumat Dis. (2015) 74:1483–7. 10.1136/annrheumdis-2014-207151 25990288

[17] Ledingham JM Snowden N Rivett A Galloway J Ide Z Firth J. Achievement of NICE quality standards for patients with new presentation of inflammatory arthritis: observations from the National Clinical Audit for Rheumatoid and Early Inflammatory Arthritis. Rheumatology (Oxford). (2017) 56:223–30. 10.1093/rheumatology/kew308 27694337 PMC5396801

